# Comparison of the Incidence of Post-intensive Care Syndrome (PICS) Between Elderly and Non-elderly Patients: A Subgroup Analysis of the Japan-PICS Study

**DOI:** 10.7759/cureus.60478

**Published:** 2024-05-17

**Authors:** Mumon Takita, Daisuke Kawakami, Toru Yoshida, Jumpei Tsukuda, Shigeki Fujitani

**Affiliations:** 1 Department of Emergency and Critical Care Medicine, St. Marianna University School of Medicine, Kawasaki, JPN; 2 Department of Intensive Care Medicine, Iizuka Hospital, Iizuka, JPN

**Keywords:** elderly, aging, japan-pics study, abcdef bundle compliance, post-intensive care syndrome (pics)

## Abstract

Aim: The aging society is expanding, and more elderly patients are admitted to intensive care units (ICUs). Elderly patients may have increased ICU mortality and are thought to have a high incidence of post-intensive care syndrome (PICS). There are few studies of PICS in the elderly. This study hypothesized that the elderly have an increased incidence of PICS compared to the non-elderly.

Methods: This is a subgroup analysis of a previous multicenter prospective observational study (Prevalence of post-intensive care syndrome among Japanese intensive care unit patients: The Japan-PICS study) conducted from April 2019 to September 2019. Ninety-six patients were included who were over 18 years old, admitted to the ICU, and expected to require mechanical ventilation for more than 48 hours. Physical component scales (PCS), mental component scales (MCS), and Short-Memory Questionnaire (SMQ) scores of included patients were compared before admission to the ICU and six months later. The diagnosis of PICS required one of the following: (1) the PCS score decreased ≧10 points, (2) the MCS score decreased ≧10 points, or (3) the SMQ score decreased by >40 points. Patients were classified as non-elderly (<65 years old) or elderly (≧65 years old), and the incidence of PICS was compared between these two groups.

Results: The non-elderly (N=27) and elderly (N=69) groups had incidences of PICS: 67% and 62% (p=0.69), respectively.

Conclusion: There is no statistically significant difference in the incidence of PICS in the non-elderly and elderly.

## Introduction

The aging of the world's population requires new considerations for medical care [1]. Japan, in particular, has a large proportion of elderly citizens. The percentage of elderly (≧65 years) in Japan reached 28% in 2019 and is projected to rise to 40% by 2060 [2]. There are diseases specifically associated with increased age as well as decreased physical function that must be considered in this patient population [3,4]. With an increase in the number of the aged in society, the percentage of elderly patients requiring acute care inevitably rises [5], and the admission rate of the elderly to intensive care units (ICUs) also rises [6].

Attention has recently been focused on the long-term well-being and quality of life of patients after discharge from intensive care [7]. Post-intensive care syndrome (PICS) is recognized as a condition that continues to impair physical, cognitive, and mental functioning after discharge from the ICU [8]. The incidence of PICS was reported to be 63.5% in a multicenter prospective observational study conducted in Japan [9] with a similar incidence of 50-70% reported in the United States [10]. A variety of factors have been cited as risk factors for the development of PICS, one of which is age [11-14].

In Japan, where the aging society is growing rapidly, it has been suggested that elderly patients admitted to the ICU have a higher incidence of PICS compared to the non-elderly. However, to the best of our knowledge, no studies have verified this hypothesis. We conducted a subgroup analysis of the Japan-PICS (J-PICS) study to investigate the incidence of PICS in elderly patients who survived to discharge from the ICU and compared it with non-elderly adults.

## Materials and methods

Design and study setting

This study is a subgroup analysis of the J-PICS study which was the first multicenter prospective observational study of PICS in Japan. A total of 14 institutions with 16 ICUs participated in this study. Details are as reported previously [9]. A schema of the J-PICS study is shown in Appendix 1. This study was approved by the Life Ethics Committee of St. Marianna University School of Medicine (authorization number: 4306) and the Ethics Committee of Kobe City Medical Center General Hospital (KCGH) (authorization number: Zn181008). Informed consent was obtained from all participants and/or their legal guardians. All methods were carried out in accordance with relevant guidelines and regulations.

Participants

Patients 18 years or older admitted to the ICU between April 1, 2019, and September 30, 2019, and who were expected to require ventilator management for at least 48 hours were enrolled in this study. Patients who required non-invasive ventilation were also included. Exclusion criteria included the following: patients with primary brain injury with consciousness or cognitive impairment (e.g., after CPA resuscitation, after severe brain injury), patients originally diagnosed with dementia, patients initially receiving mechanical ventilation, patients with terminal malignancies, patients receiving limited treatment, patients who were not expected to survive for more than 24 hours, patients readmitted to the ICU, patients who had no family, friends, or caregivers to know their condition, patients who do not use the Japanese language, patients with difficult follow-up or who could not be followed up after six months (e.g., unreturned/unanswered questionnaires), and patients who did not agree to participate in the study.

Data collection

The following items were collected as baseline information for each patient including age, gender, body mass index (BMI), Acute Physiology and Chronic Health Evaluation (APACHE) II score, Sequential Organ Failure Assessment (SOFA) score, Charlson comorbidity index, clinical frailty scale (CFS), do not attempt resuscitation status on admission, educational level, employment status, marital status, residential living status before admission, history of treatment with benzodiazepines and steroids, source of admission to ICU, and primary diagnosis at the time of ICU admission, adult respiratory distress syndrome, presence of sepsis, and management in the ICU (such as use of catecholamines and use of renal replacement therapy). In addition, ABCDEF bundle compliance checks (Appendix 2) are performed 1-4 days after admission to the ICU.

The ABCDEF bundle is a bundle devised for the prevention of PICS [15] and includes assessment, prevention, and management of pain; both spontaneous awakening trials (SATs) and spontaneous breathing trials (SBTs); choice of sedation/analgesia; delirium monitoring and management; early mobility and exercise; and family engagement and empowerment. This tool has been shown to improve mortality, duration of ventilation, and duration of delirium and coma-free status by increasing compliance with a management bundle [16,17]. ABCDEF bundle compliance was calculated as the mean value over three days (Appendix 3). When patients had contraindications for each bundle compliance and did not meet the criteria for each bundle, compliance with each bundle was regarded as having been met.

Follow-up was conducted for six months following ICU admission, and mortality (ICU, hospital, six months), ICU and hospital length of stay, duration of mechanical ventilation, occurrence of delirium, and disposition status (discharge to home or not) were investigated. Physical, mental, and cognitive functions were compared before and six months after ICU admission using SF-36 and Short-Memory Questionnaire (SMQ). SF-36 consists of the physical component scale (PCS), an assessment of physical functioning, and the mental component scale (MCS), an assessment of mental functioning, each rated with a full score of 100 points, with higher values judged to be better. Cognitive functions were evaluated using SMQ. The SMQ is also in the form of a questionnaire with 12 questions to answer, and scores range from 4 to 46, with scores below 40 considered cognitive impairment. These questionnaires were completed by the patient's healthcare decision-making proxy and were scored at ICU admission and six months after ICU admission, respectively, and the scores were compared. Questionnaires at six months were mailed to patients' homes.

Interventions and comparisons

Patients enrolled in the J-PICS study were divided into two groups, elderly (≧65 years) and non-elderly (<65 years), and compared in terms of PICS incidence, pre-admission baseline, ICU length of stay, hospital length of stay, duration of ventilatory support, ABCDEF bundle compliance, delirium incidence, and rate of discharge to home.

PICS was defined as fulfillment of any of the following (1) to (3) items in a survey conducted six months after ICU admission: (1) if the PCS declines by 10 points or more, (2) if the MCS declines by 10 points or more, or (3) if the SMQ declines and is less than 40 points.

Statistical analysis

Univariate analysis was performed using the Wilcoxon rank-sum test for continuous variables, and the chi-squared test or Fisher's exact test was used for categorical variables, if the number in a category was less than 10. Multivariate logistic regression model was used to identify factors associated with the occurrence of PICS. Multivariate analysis included the following variables as confounding factors: age≧65, ABCDEF bundle, education level, and CFS. Statistical significance was set at P<0.05. Statistical analyses were performed using JMPR 13.0.0 (SAS Institute Inc., Cary, NC, USA).

## Results

**Figure 1:**
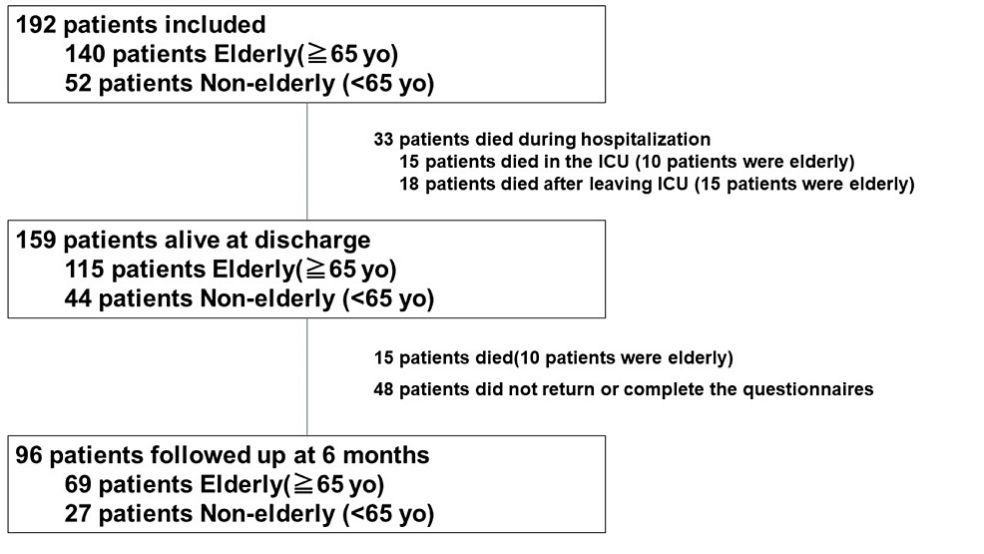
Flowchart of included patients One hundred and ninety-two patients were included in the J-PICS study, and 96 underwent follow-up. Of these 96 patients, 69 were elderly (≧65 years old), and 27 were non-elderly (<65 years old).

A total of 192 patients were included in the J-PICS study, of whom 96 were ultimately followed up (Figure 1).

Of these 96 patients, 69 were ≥65 years old (elderly), and 27 were <65 years old (non-elderly). Baseline characteristics and management in the ICU were compared between elderly and non-elderly patients (Table 1). Significant differences were observed between the elderly and non-elderly for Charlson comorbidity index, CFS, educational level, employment status, marital status, residential living status, history of treatment with benzodiazepines, primary diagnosis at the time of admission to the ICU, and ABCDEF bundle compliance.

**Table 1:** Patient characteristics Data are presented as median (interquartile range) or number (percentage). APACHE: Acute Physiology and Chronic Health Evaluation; SOFA: Sequential Organ Failure Assessment; ICU: intensive care unit

	Age<65 (N=27)	Age≧65 (N=69)	P-value	Overall (N=96)
Male, N(%)	19 (70.3)	49 (71.0)	1.00	68 (70.8)
Age	51 (41-58)	78 (72.5-82)	<0.01	74 (63.3-81)
Body mass index	23.2 (19.8-25.7)	22.4 (19.5-24.9)	0.32	22.5 (19.6-25.0)
APACHE Ⅱ score	19 (13-25)	21 (17-25)	0.13	20 (16-25)
SOFA score	8 (5-11)	8 (6-11)	0.85	8 (6-11)
Charlson comorbidity index	0 (0-1)	1 (0-2)	0.01	1 (0-2)
Clinical frailty scale	3 (1-3)	3 (2-4)	0.02	3 (2-4)
Do not attempt resuscitation, N(%)	1 (3.7)	2 (2.9)	1.00	3 (3.1)
Education level, N(%)				
≦9 years	1 (3.7)	17 (25.8)	0.02	18 (19.4)
>9 years	26 (96.3)	49 (74.2)		75 (80.7)
Employment status, N(%)			<0.01	
Employed or self-employed	15 (55.6)	17 (25)		32 (33.7)
Unemployed	6 (22.2)	8 (11.8)		14 (14.7)
Housework	3 (11.1)	7 (10.3)		10 (10.5)
Retired	3 (11.1)	34 (50.0)		37 (39.0)
Marital status, N(%)			<0.01	
Married	11 (40.7)	53 (76.8)		64 (66.7)
Separated or divorced	5 (18.5)	2 (2.90)		7 (7.29)
Widowed	0 (0)	14 (20.3)		14 (14.6)
Unmarried	11 (40.7)	0 (0)		11 (11.5)
Patient's residential living status, N(%)			<0.01	
Lived alone at home	9 (33.3)	6 (8.7)		15 (15.6)
Lived with someone else	18 (66.7)	62 (89.9)		80 (83.3)
Nursing home	0 (0)	1 (1.45)		1 (1.04)
History of treatment with benzodiazepines, N(%)	7 (25.9)	3 (4.35)	<0.01	10 (10.4)
History of treatment with steroids, N(%)	2 (7.41)	4 (5.80)	1.00	6 (6.25)
Source of admission to ICU, N(%)			0.42	
Emergency department	11 (40.7)	32 (46.4)		43 (44.8)
Hospital floor	4 (14.8)	17 (24.6)		21 (21.9)
Another hospital	0 (0)	1 (1.45)		1 (1.04)
Operating room (elective)	0 (0)	1 (1.45)		1 (1.04)
Operating room (emergency)	12 (44.4)	18 (26.1)		30 (31.3)
Primary diagnosis at the time of admission in ICU, N(%)			0.002	
Cardiogenic	3 (11.1)	12 (17.4)		15 (15.6)
Acute respiratory failure	4 (14.8)	30 (43.5)		34 (35.4)
Infection	5 (18.5)	16 (23.2)		21 (21.9)
Trauma	7 (25.9)	4 (5.80)		11 (11.5)
Others	8 (29.6)	7 (10.1)		15 (15.6)
Acute respiratory distress syndrome, N(%)	5 (18.5)	12 (17.4)	0.90	17 (17.7)
Sepsis, N(%)	8 (29.6)	23 (33.3)	0.73	31 (32.3)
ICU management				
Inotrope/vasopressor	20 (74.1)	57 (82.6)	0.89	77 (80.2)
Paralytic agent	5 (18.5)	7 (10.1)	0.26	12 (12.5)
Renal replacement therapy in ICU	3 (11.1)	9 (13.0)	0.80	12 (12.5)
Extracorporeal membrane oxygenation	2 (7.41)	2 (2.90)	0.32	4 (4.17)
Intra-aortic balloon pump	1 (3.70)	2 (2.90)	0.84	3 (3.13)
Tracheostomy	5 (18.5)	8 (11.6)	0.37	13 (13.5)
ICU length of stay (days)	7 (4-15)	7 (5-11.5)	0.96	7 (5-12)
Delirium, N(%)	5 (18.5)	26 (37.7)	0.09	31 (32.3)
ABCDEF bundle compliance	0.67 (0.58-0.75)	0.72 (0.67-0.82)	0.01	0.72 (0.64-0.81)

Table 2 shows the results of follow-up after discharge from the ICU. There were no significant differences in the incidence of PICS (67% in the non-elderly vs. 62% in the elderly, p=0.69). There were also no significant differences in hospital length of stay (non-elderly 33 (18-61) vs. elderly 34 (20.5-61.5), p=0.56), days of mechanical ventilation (non-elderly 5 (3-13) vs. elderly 5 (3-6.5), p=0.35), and discharge to home rates (non-elderly 51.9% vs. elderly 46.4%, p=0.63). There were no significant differences in mortality (ICU, hospital, or six months, as shown in Appendix 4) between the two groups: ICU mortality (non-elderly 9.6% vs. elderly 7.1%, p=0.56), hospital mortality (non-elderly 15.4% vs. elderly 17.9%, p=0.83), and six-month mortality (non-elderly 23% vs. elderly 32%, p=0.19).

**Table 2:** Outcomes for patients age<65 and age≧65 PICS: post-intensive care syndrome

	Age<65 (N=27)	Age≧65 (N=69)	P-value	OR (95% CI)	Overall (N=96)
PICS N(%)	18 (67)	43 (62)	0.69	0.83 (0.32-2.10)	61 (64)
Discharge home N(%)	14 (51.9)	32 (46.4)	0.63	0.83 (0.33-1.96)	46 (47.9)
Hospital length of stay (days)	33 (18-61)	34 (20.5-61.5)	0.56	-	33.5 (18.3-61)
Days of mechanical ventilation	5 (3-13)	5 (3-6.5)	0.35	-	5 (3-8.75)

The breakdown of PICS impairments was also investigated, and the comparative findings between the elderly and non-elderly are shown in Table 3. There were no significant differences in the breakdown of physical impairment (non-elderly 67% vs. elderly 44%, p=0.16), mental impairment (non-elderly 27.8% vs. elderly 20.9%, p=0.74), cognitive impairment (non-elderly 61.1% vs. elderly 58.1%, p=1.00), two or more functional disorders (non-elderly 50.0% vs. elderly 23.3%, p=0.77), and incidence of PICS.

**Table 3:** Impairments of non-elderly and elderly patients

	Age<65 (N=18)	Age≧65 (N=43)	P-value	Overall (N=61)
Physical impairment N(%)	12 (66.7)	19 (44.2)	0.16	31 (50.8)
Mental impairment N(%)	5 (27.8)	9 (20.9)	0.74	14 (23)
Cognitive impairment N(%)	11 (61.1)	25 (58.1)	1.00	36 (59)
Two or more functional disorders	9 (50.0)	10 (23.3)	0.07	19 (31.2)

With PICS onset as the outcome, multivariate analysis was performed, and the results are shown in Table 4. Being elderly (age ≧65) was not associated with the incidence of PICS (OR: 1.51 (95% CI 0.53-4.26)).

**Table 4:** Multivariate logistic regression analysis for PICS PICS: post-intensive care syndrome

	OR (95% CI)	P-value
Age ≧65 years	1.51 (0.53-4.26)	0.44
ABCDEF bundle compliance	0.33 (0.01-10.1)	0.52
≦9 years of formal education	3.65 (0.92-14.4)	0.06
Clinical frailty scale	1.01 (0.73-1.40)	0.94

We compared the incidence of PICS among the elderly (65 years or older), with subgroups by age, at 75, 80, and 85 years, and there were no significant differences (Appendix 5).

## Discussion

Key finding of this study

Although being elderly has been considered by some to be a risk factor for developing PICS, there is no significant difference in the incidence of PICS between elderly and non-elderly patients in the present study. To the best of our knowledge, there are no previous reports comparing the incidence of PICS between the elderly and non-elderly.

The lack of a difference in the incidence of PICS in the elderly and non-elderly

Lee et al. discussed two separate factors that contribute to the development of PICS, personal and ICU-related, in their systematic review [18]. They concluded that older age, female gender, and pre-existing psychological conditions as important patient factors and severity, negative ICU experience, and delirium as ICU-related factors have a strong influence on the development of PICS [18]. Kawakami et al. reported in the J-PICS study that a lower education level (only mandatory education) was associated with the development of PICS [9].

Risk factors for the development of PICS for elderly and non-elderly patients were compared, focusing on patient background in the present study. No significant differences were found between elderly and non-elderly patients with respect to gender, severity of illness, or delirium. No data were obtained on pre-existing psychological conditions or negative ICU experiences in the present study. With regard to education, the elderly group has a higher proportion of people at a lower education level (only mandatory education; period of education with less than nine years). Thus, patient background with regard to risk factors for the development of PICS was compared between elderly and non-elderly patients, and there are no differences in risk factors for developing PICS in non-elderly patients. Lee et al. emphasized that patient factors cannot be changed by medical interventions, but ICU-related factors (especially negative ICU experiences and delirium) can be changed by interventions [18]. Examining compliance with the ABCDEF bundle, which is designed to prevent PICS [15], the data shows that the level of compliance with the ABCDEF bundle is significantly higher in the elderly group. This effect may have been influenced by ICU staff having the impression that the elderly have a greater risk of developing PICS. ICU staff may have been more diligent in performing the ABCDEF bundle in the elderly, so the PICS incidence may have been lower in the elderly than it might have otherwise been. In fact, the incidence of delirium was not significantly different between elderly and non-elderly patients. It is also reasonable to suggest that a higher ABCDEF bundle compliance rate reduces negative ICU experiences. However, these are only speculations, since multivariate analysis in our study did not suggest a causal relationship between ABCDEF bundle compliance and incidence of PICS. Since the incidence of PICS is not significantly different between the elderly and the non-elderly in the present study, it may be important that dementia is an exclusion criterion for this study. Since, in general, the incidence of dementia is higher in the elderly, it is possible that excluding patients with dementia in the present study may have affected the incidence of PICS in the elderly group.

Focusing on the population included in this study, the elderly group was relatively healthy with a median CFS of 3 and a median BMI of 22.4. CFS is suitable for assessing a patient's baseline frailty and is a score from 1 to 9, with higher scores indicating poorer physical function [19]. This may partially explain why mortality and the incidence of PICS did not differ between elderly and non-elderly in the present study.

Focusing on the age of the elderly included in the present study, the median age was 78 (72.5-82). A review of previous studies investigating long-term functional prognosis in the elderly showed that many included elderly were over 80 years old [20]. The relatively young age of the elderly included in the present study may also be a reason for the lack of a difference in the incidence of PICS between the elderly and the non-elderly.

PICS in the elderly

Although this study focused on the incidence of PICS in the elderly and non-elderly, attention should also be paid to the incidence of PICS among the elderly including stratification by age groups. In developed countries, the number of very elderly admitted to the ICU is increasing [6]. Therefore, it is necessary to subdivide the elderly further by age group and compare the incidence of PICS. Past studies have evaluated ADL changes among elderly who were discharged alive from the ICU [21]. Chelluri et al. investigated mortality and ADLs one year after ICU discharge among the elderly (≥65) and compared them to 65-74-year-old patients (N=43) and patients aged ≥75 years (N=54) and found no significant differences [21]. We also investigated whether there were differences in the incidence of PICS among the elderly (Appendix 5) but found no significant differences in the incidence of PICS, similar to previous studies.

Strengths and limitations

The present study has acknowledged strengths. First, this is a multicenter study. Second, all items that contribute to PICS in the elderly were evaluated. Existing studies have focused only on individual items related to PICS, such as ADLs and cognitive function [22-25]. The present study is epidemiologically novel because it evaluates each of the three items (physical function, mental function, and cognitive function) as defined in PICS and compares them using quantitative scores before admission to the ICU with evaluations six months after admission. Third, the incidence of PICS is compared between elderly and non-elderly patients. To the best of our knowledge, no previous study has compared the incidence of PICS between the elderly and non-elderly, contributing to the novelty of the present study.

The present study also has acknowledged limitations. First, there is age bias in the patient group. The median age of the elderly included in this study is 77 years, and there were very few patients over 80 years old, which has been more extensively included in previous studies [23,25], which may have affected the results. Second, there are differences in the ability of patients to perform ADLs among the patient groups in the present study. Elderly patients showed a median pre-admission CFS of 3 and a median BMI of 22.4, suggesting that patients in this study had a relatively good ability to perform ADLs and good nutritional status. These factors may have affected mortality and the incidence of PICS. Third, there is a wide range of 95% confidence intervals for odds ratios of PICS incidence between elderly and non-elderly patients (OR 0.83; 95% CI 0.32-2.10). A larger sample size could have produced different results. Finally, the follow-up period is relatively short at six months. Some reports have longer follow-ups, such as one year after ICU discharge [22-25]. If followed for one year after ICU admission, both mortality and PICS incidence would likely have been higher among the elderly. This may have contributed to the difference in the incidence of PICS in the elderly and non-elderly.

## Conclusions

Older age is generally considered a risk factor for the development of PICS. However, in this subgroup analysis of data from the J-PICS study, there is no significant difference in the incidence of PICS in non-elderly and elderly patients. To the best of our knowledge, there are no previous reports comparing the incidence of PICS between the elderly and non-elderly.

In this study, included elderly were relatively healthy (median CFS of 3 and a median BMI of 22.4), and ABCDEF bundle (which is designed to prevent PICS) compliance in the elderly is higher than the non-elderly. These factors could have influenced the outcome.

## Figures and Tables

**Figure 2:**
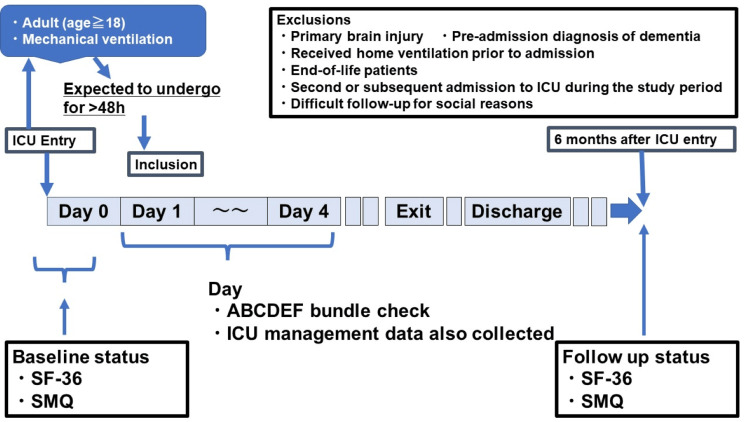
A schema of the Japan-PICS study PICS: post-intensive care syndrome; ICU: intensive care unit; SMQ: Short-Memory Questionnaire

**Figure 3:**
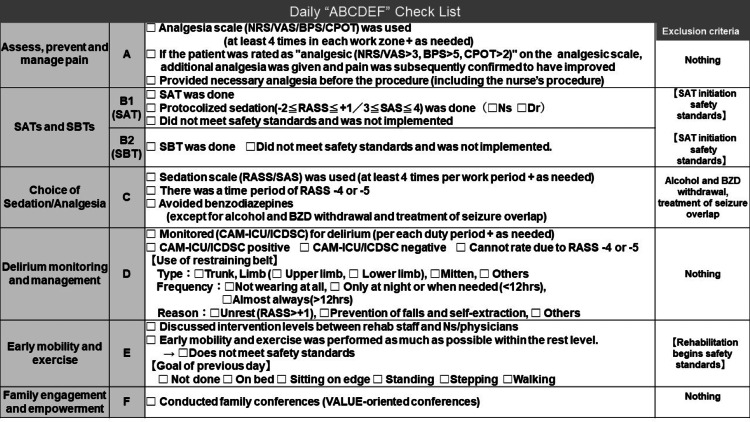
Daily ABCDEF checklist SAT/protocolized sedation initiation safety standards: Not using continuous sedation medications for active seizure control Not requiring the use of sedatives for alcohol withdrawal Not using muscle relaxants RASS ≤+2 No myocardial ischemic events within 24 hours No elevated ICP (ICP<20) Not using sedatives to control ICP Not using ECMO Not under open abdominal/thoracotomy management SBT initiation safety standards: Recovering from the primary illness that required a ventilator Able to maintain SpO2 ≥88% under FiO2 ≤50% and PEEP ≤8 cmH2O Hemodynamically stable (no need to increase catecholamine dose) No active ischemic events No use of muscle relaxants No effort breathing Minute ventilation ≤15 L/min RASS +1 to -2 Rehabilitation begins safety standards: 2>RASS>-3 PEEP <10 cmH2O and FiO2 <60 No new catecholamine initiation or dose increase within two hours No new myocardial infarction within two hours No treatment requiring rest restriction (open abdomen/open chest/ICP monitoring during drainage/IABP/ECMO) No contraindications to bed rest (e.g., unstable fracture) SAT: Spontaneous Awakening Trial; RASS: Richmond Agitation-Sedation Scale; ICP: intracranial pressure; ECMO: extracorporeal membrane oxygenation; SBT: Spontaneous Breathing Trial; SpO2: oxygen saturation; FiO2: fraction of inspired oxygen; PEEP: positive end-expiratory pressure; IABP: intra-aortic balloon pump

## Appendices

Appendix 1

Appendix 2

Appendix 3

**Table 5:** ABCDEF bundle check NRS: Number Rating Scale; VAS: Visual Analogue Scale; BPS: Behavioral Pain Scale; CPOT: Critical Care Pain Observation Tool; SAT: Spontaneous Awakening Trial; SBT: Spontaneous Breathing Trial; RASS: Richmond Agitation-Sedation Scale; SAS: Sedation-Agitation Scale; CAM-ICU: Confusion Assessment Method for the ICU; ICDSC: Intensive Care Delirium Screening Checklist

Item	Evaluation
A-1	Analgesic scale (NRS/VAS/BPS/CPOT) was used
A-2	When the pain scale evaluated as painful (NRS/VAS>3, BPS>5, CPOT>2), analgesics were administered, and pain relief was subsequently observed
A-3	Analgesics were administered before treatment (including nurse's treatment), if necessary
B-1	SAT was conducted (including patients where safety standards are not met and implementation is not carried out)
B-2	SBT was conducted (including patients where safety standards are not met and implementation is not carried out)
C-1	Sedative scale (RASS/SAS) was used
C-2	Patients had occasionally a deep sedation (RASS -4 or -5)
C-3	Use of benzodiazepine (BZD) drugs was avoided
D-1	Delirium was monitored by using CAM-ICU or ICDSC
E-1	Nurse, physicians, and rehabilitation staff discussed intervention levels of rehabilitation
E-2	The maximum possible early out-of-bed rehabilitation was performed within the range of rest (including cases where safety standards were not met and could not be performed)
F	Family conference held
Calculate "number of 12 items/total 12 items" for each of days 2-4. Average of 3 days (length of stay in case of early exit) = Bundle compliance rate(impl_all)	

Appendix 4

**Table 6:** Comparison of patients aged <65 and aged ≧65 Data are presented as median (interquartile range) or number (percentage). a: missed nine patients, b: missed 21 patients, c: missed 30 patients BMI: body mass index; APACHE: Acute Physiology and Chronic Health Evaluation; SOFA: Sequential Organ Failure Assessment; ICU: intensive care unit; ARDS: acute respiratory distress syndrome; ECMO: extracorporeal membrane oxygenation; IABP: intra-aortic balloon pump; PICS: post-intensive care syndrome

	Age<65 (N=52)	Age≧65 (N=140)	P-value	Overall (N=192)
Male, N(%)	34 (65.4)	91 (65.0)	0.96	125 (65.1)
BMI	23.6 (20.1-27.5)	21.6 (19.4-24.1)	0.02	21.9 (19.6-25.0)
APACHE Ⅱ score	20.5 (14-27.8)	23 (19.3-28)	0.05	21.9 (19.5-25.0)
SOFA score	8 (5-11)	8 (6-11)	0.85	8 (6-11)
Charlson comorbidity index	0 (0-3)	1 (0-3)	0.05	1 (0-3)
Clinical frailty scale	3 (2-4 )	4 (3-5)	<0.01	3 (3-4)
Do not attempt resuscitation, N(%)	3 (5.8)	12 (8.6)	0.76	15 (7.8)
Educational level, N(%)				
≦9 years	7 (14.3)	42 (32.8)	0.01	49 (27.7)
>9 years	42 (85.7)	86 (67.2)		128 (72.3)
Employment status, N(%)			<0.01	
Student	2 (3.9)	0 (0)		2 (1.1)
Employed or self-employed	26 (51)	32 (23.4)		58 (30.9)
Unemployed	14 (27.5)	27 (19.7)		41 (21.8)
Housework	5 (9.8)	16 (11.7)		21 (11.2)
Retired	4 (7.8)	60 (43.8)		64 (34.0)
Marital status, N(%)			<0.01	
Married	21 (40.4)	98 (70)		119 (62.0)
Separated or divorced	9 (17.3)	7 (5)		16 (8.3)
Widowed	2 (3.9)	35 (25)		37 (19.3)
Unmarried	20 (38.5)	0 (0)		20 (10.4)
Patient's residential living status, N(%)			<0.01	
Lived alone home	18 (34.6)	15 (10.7)		33 (17.2)
Lived with someone else	34 (65.4)	118 (84.3)		152 (79.2)
Nursing home	0 (0)	7 (5.0)		7 (3.7)
History of treatment with benzodiazepines, N(%)	12 (23.1)	6 (4.3)	<0.01	18 (9.4)
History of treatment with steroids, N(%)	4 (7.7)	21 (15)	0.18	25 (13)
Source of admission to ICU, N(%)			0.26	
Emergency department	24 (46.2)	71 (50.7)		95 (49.5)
Hospital floor	9 (17.3)	36 (25.7)		45 (23.4)
Another hospital	2 (3.9)	1 (0.7)		3 (1.6)
Operating room (elective)	1 (1.9)	3 (2.1)		4 (2.1)
Operating room (emergency)	16 (30.8)	29 (20.7)		45 (23.4)
Primary diagnosis at the time of admission in ICU, N(%)			<0.01	
Cardiogenic	4 (7.7)	21 (15)		25 (13.0)
Acute respiratory failure	7 (13.5)	60 (42.9)		67 (34.9)
Infection	14 (26.9)	42 (30)		56 (29.2)
Trauma	10 (19.2)	6 (4.3)		16 (8.3)
Others	17 (32.7)	11 (7.9)		28 (14.6)
ARDS, N(%)	9 (17.3)	26 (18.6)	0.84	35 (18.2)
Sepsis, N(%)	20 (38.5)	59 (42.1)	0.65	79 (41.2)
Management in ICU				
Inotrope/vasopressor	39 (75)	109 (77.9)	0.68	148 (77)
Paralytic agent	8 (15.4)	16 (11.4)	0.46	24 (12.5)
Renal replacement therapy in ICU	15 (28.9)	25 (17.9)	0.10	40 (20.8)
ECMO in ICU	3 (5.8)	4 (2.9)	0.34	7 (3.7)
IABP in ICU	1 (1.9)	5 (3.6)	0.34	6 (3.1)
Tracheostomy	9 (17.3)	20 (14.3)	0.27	29 (15.1)
Mortality				
ICU mortality, N(%)	5(9.6)	10(7.1)	0.56	15 (7.8)
Hospital mortality, N(%)	8 (15.4)	25 (17.9)	0.83	33 (17.2)
Six-month mortality, N(%)	10 (23.3)^a^	38 (31.9)^b^	0.19	48 (29.6)^c^
ICU length of stay (days)	8 (5-14.8)	7 (5-13)	0.67	7 (5-14)
Hospital length of stay (days)	36.5 (16.5-59.5)	32.0 (19.3-63.5)	0.49	33.5 (19-61.8)
Days of mechanical ventilation	6 (3-13.8)	5 (3-10)	0.19	6 (3-11)
PICS, N(%)	18 (66.7)	43 (62.3)	0.81	61 (63.5)
ABCDEF bundle compliance	0.67 (0.58-0.75)	0.72 (0.67-0.82)	0.01	
Delirium, N(%)	10 (19.2)	51 (36.4)	0.03	61 (31.8)
Discharged from hospital among survivors, N(%)				N=159
Home	23 (44.2)	48 (34.3)	0.17	71 (37.0)
Another facility	21 (40.4)	66 (57.4)	0.40	87 (45.3)
Nursing home		1 (0.7)		1 (0.5)

Appendix 5

**Table 7:** Incidence of PICS according to age Data are presented as number of PICS/total number (percentage); all ages are in years. PICS: post-intensive care syndrome

Age	Incidence of PICS	P-value
Age<65 vs. ≧65	18/27 (67) vs. 43/69 (62)	0.69
65≦age<75 vs. 75≦age	15/23 (65) vs. 28/46 (60)	0.80
65≦age<80 vs. 80≦age	25/43 (58) vs. 18/26 (69)	0.45
65≦age<85 vs. 85≦age	36/59 (61) vs. 7/10 (70)	0.73
